# Mechanisms of ER Stress-Mediated Mitochondrial Membrane Permeabilization

**DOI:** 10.1155/2010/170215

**Published:** 2010-02-07

**Authors:** Sanjeev Gupta, Lorraine Cuffe, Eva Szegezdi, Susan E. Logue, Catherine Neary, Sandra Healy, Afshin Samali

**Affiliations:** Apoptosis Research Centre, School of Natural Sciences, National University of Ireland, Galway, Ireland

## Abstract

During apoptosis, the process of mitochondrial outer membrane permeabilization (MOMP) represents a point-of-no-return as it commits the cell to death. Here we have assessed the role of caspases, Bcl-2 family members and the mitochondrial permeability transition pore on ER stress-induced MOMP and subsequent cell death. Induction of ER stress leads to upregulation of several genes such as Grp78, Edem1, Erp72, Atf4, Wars, Herp, p58ipk, and ERdj4 and leads to caspase activation, release of mitochondrial intermembrane proteins and dissipation of mitochondrial transmembrane potential (ΔΨm). Mouse embryonic fibroblasts (MEFs) from caspase-9, -2 and, -3 knock-out mice were resistant to ER stress-induced apoptosis which correlated with decreased processing of pro-caspase-3 and -9. Furthermore, pretreatment of cells with caspase inhibitors (Boc-D.fmk and DEVD.fmk) attenuated ER stress-induced loss of ΔΨm. However, only deficiency of caspase-9 and -2 could prevent ER stress-mediated loss of ΔΨm. Bcl-2 overexpression or pretreatment of cells with the cell permeable BH4 domain (BH4-Tat) or the mitochondrial permeability transition pore inhibitors, bongkrekic acid or cyclosporine A, attenuated the ER stress-induced loss of ΔΨm. These data suggest a role for caspase-9 and -2, Bcl-2 family members and the mitochondrial permeability transition pore in loss of mitochondrial membrane potential during ER stress-induced apoptosis.

## 1. Introduction

The endoplasmic reticulum (ER) is a cytosolic membrane bound network connected to the nucleus, mitochondria, and the plasma membrane. Membrane and secreted proteins are targeted to the ER for folding and posttranslational modification [1, 2]. In addition, the ER is the primary storage organelle for intracellular Ca^2+^, thereby the main regulator of cellular Ca^2+^ homeostasis. Given its central role in protein folding and its influence on Ca^2+^-mediated signaling pathways, disruption of the ER homeostasis, also called ER stress, has severe consequences for the cell [1, 2]. A number of pathophysiological conditions are associated with ER stress, including stroke, ischemia, hyperhomocystinemia, diabetes, viral infections, and mutations that impair protein folding [3, 4]. To combat the deleterious effects of ER stress, the cell has evolved a variety of protective strategies collectively known as the Unfolded Protein Response (UPR). This concerted and complex cellular response is initiated by three molecules, PERK (PKR-like ER kinase), ATF6 (activated transcription factor-6), and IRE1 (Inositol requiring enzyme 1) [1]. 

The UPR attempts to reduce the protein load on the ER and increase the folding capacity of the ER [5]. However, unresolved ER stress results in the activation of apoptosis. The exact mechanism involved in transition of the UPR from a protective to an apoptotic response is not clearly understood, but it does appear to be dependent on cysteinyl aspartate proteases of the caspase family and the proteins of Bcl-2 family [6, 7]. Several studies have reported the involvement of initiator caspase-2, -8, and -9 [8, 9] and effector caspase-3 and -7 in ER stress-induced apoptosis [10]. It has also been suggested that caspase-12 acts as an initiator caspase during ER stress-induced apoptosis [11, 12]. However, a significant role for caspase-12 in ER stress-induced apoptosis has not been supported by the majority of the literature (reviewed in [13]). For example, caspase-12-deficient murine P19 embryonic carcinoma cells do not exhibit altered levels of tunicamycin-induced DNA fragmentation [8]. Also ER stress-induced cell death is unaffected by an absence of caspase-12 in B16/B16 melanoma cells [14] or in MEFs isolated from caspase-12 deficient mice [15]. Furthermore, in humans, a single nucleotide polymorphism in caspase-12 results in the synthesis of a truncated protein, lacking enzymatic activity [16]. Recent reports implicate the involvement of mitochondria in ER stress-induced apoptosis [10]. Release of cytochrome *c* from mitochondria during ER stress-induced apoptosis has been suggested to be mediated by mitochondrial permeability transition (MPT) [17, 18]. The molecular mechanism of the mitochondrial membrane depolarisation and the release of cytochrome *c* are well studied in various types of cellular stresses, and two mechanistically different models have been proposed [19]. The first one is controlled by proteins of the Bcl-2 family, while the second one involves a high conductance ion channel, the permeability transition pore (PTP) [20]. The role of the Bcl-2 family in ER stress-induced apoptosis is emphasized by concurrent repression of Bcl-2 and upregulation of Bim by the transcription factor, CHOP, a key determinant of ER stress-induced apoptosis [21, 22]. Furthermore, expression of the BH3 only proteins, Noxa and Puma, has been reported to be upregulated in MEFs undergoing ER stress-induced apoptosis [23]. Bcl-2 family members are known to localize both to the ER and the mitochondria, where they may act to regulate the signaling pathways that promote the opening of the PTP [19, 24]. For example, Bax and Bak can directly bind to the PTP and may act to induce MPT. They may also cause MPT by enhancing Ca^2+^ release from the ER [25]. On the other hand, when the death antagonists, such as Bcl-2 and Bcl-x_L_ bind to the PTP, they prevent the opening of the channel in response to many apoptotic signals [19]. The two models of cytochrome *c* release are not independent. The cytochrome *c*-dependent apoptotic pathway activated by ER-mitochondria crosstalk seems to play an essential role in the ER stress-mediated cell death [26].

The ER and mitochondria are in close contact which supports communication between these two organelles, including synthesis and transfer of lipids, and the exchange of Ca^2+^, that regulates ER chaperones, mitochondrial ATP production and apoptosis [27]. Here we have determined the role of caspases, Bcl-2 family members, and PTP on the mitochondrial changes associated with ER-induced apoptosis. Our results show that ER stress-induced apoptosis involves loss of ΔΨm that is dependent on caspases and regulated by Bcl-2 family members and the mitochondrial PTP. 

## 2. Materials and Methods

### 2.1. Cell Culture and Treatments

The rat neonatal cardiomyocyte-derived cell line H9c2 (ATCC) was cultured in Dulbecco's modified Eagle's medium supplemented with 10% fetal bovine serum, 50 U/ml penicillin and 5 mg/ml streptomycin. To induce ER stress, cells were treated with 2 *μ*M thapsigargin (Tg), 2 *μ*g/ml Tm, or 2 *μ*g/ml BFA for the indicated time periods. The broad range caspase inhibitors Boc-D.fmk and DEVD.fmk (Enzyme Systems Products) were used at a concentration of 20 *μ*M, while BH4-Tat peptide (Calbiochem) was used at 200 nM. To study MPT, bongkrekic acid (BA, Calbiochem) was dissolved in 2N NH_4_OH and used at 10 *μ*M final concentration, cyclosporine A (CsA) at 2 *μ*M, aristolochic acid (ArA, Calbiochem) at 25 *μ*M, dissolved in DMSO. All reagents were from Sigma-Aldrich unless otherwise stated.

### 2.2. RNA Extraction and Real Time RT-PCR

Total RNA was isolated using Trizol (Invitrogen) according to the manufacturer's instructions. Reverse transcription (RT) was carried out with 2 *μ*g RNA and Oligo dT (Invitrogen) using 20 U Superscript II Reverse Transcriptase (Invitrogen). For real-time PCR experiments, cDNA products were mixed with 2 ×TaqMan master mix and 20 ×TaqMan Gene Expression Assays (Applied Biosystems) and subjected to 40 cycles of PCR in StepOnePlus instrument (Applied Biosystems). Relative expression was evaluated with ΔΔ*C*_*T*_ method.

### 2.3. Plasmid Transfections and Generation of Stable Clones

H9c2 cells grown to 85% confluency in 6 well plates were cotransfected with 1.5 *μ*g of Bcl-2 (a kind gift from Prof., Stanley Korsmeyer, Howard Hughes Medical Institute, Boston, Massachusetts, USA) and 0.15 *μ*g of pPUR puromycin resistance vector (Clontech) using Effectene transfection reagent (Qiagen) as per the manufacturer's instructions. Puromycin (5 *μ*g/ml) was added 48 hours posttransfection to select stably transfected cells. Cells were cultured for three weeks to generate pooled transfectants.

### 2.4. Cell Viability Assay

Viability of cells after treatment was analysed by MTT assay. After treatment of cells with appropriate drugs, 1 mg/ml concentration of MTT (3-(4, 5-dimethylthiazol-2-yl)-2, 5-diphenyl tetrazolium bromide) was added to the wells and incubated at 37°C for 3 hours. The reaction was stopped with a stop mix containing 20% SDS in 40% dimethylformamide. The color intensity is measured at 550 nm, and percentage cell viability is calculated using the untreated samples as 100%. 

### 2.5. Annexin V Staining 

Externalization of phosphatidylserine (PS) to the outer leaflet of the plasma membrane of apoptotic cells was assessed with annexin V-FITC. Briefly, cells were collected by centrifugation at 350 g, washed once in ice-cold calcium buffer (10 mM HEPES/NaOH, pH 7.4, 140 mM NaCl, 2.5 mM CaCl_2_), and incubated with annexin V-FITC or with annexin V-PE for 15 minutes on ice. A wash step in calcium buffer was carried out prior to acquisition on a FACSCalibur flow cytometer (Becton Dickinson).

### 2.6. Western Blot Analysis

Protein samples (15–20 *μ*g protein per lane) were resolved on 10% SDS-PAGE gels and electrophoretically transferred to nitrocellulose membranes. After blocking in 5% nonfat milk and 0.05% Tween 20 in PBS, blots were incubated with antibodies to KDEL (1:1,000, StressGen), caspase 3 (1:1,000, Cell Signaling Technology), CHOP (Santa Cruz, 1:2,000), PKC*δ* (Santa Cruz, 1:1,000), caspase 7, and 9 (1:1,000; Cell Signaling Technologies) and caspase 12 (1:1,500; Cell Signaling Technologies). The appropriate HRP-conjugated secondary antibodies (Pierce) were used at a 1:2,000 for antibodies from Cell Signaling technologies and at a 1:10,000 dilution for all other antibodies. Protein bands were detected with Super Signal Ultra Chemilumiescent Substrate (Pierce) on X-ray film (Agfa).

### 2.7. Cell Morphology 

Cells were seeded onto 18 mm coverslips at a density of 4 × 10^4^ cells/ml. After treatment with thapsigargin, cells were fixed in methanol for 5 minutes at room temperature and stained with Harris hematoxylin and Eosin Y as previously described [28]. 

### 2.8. Rapid Preparation of Cytosolic Fraction

Cell fractions were prepared as described previously [29]. Briefly, cells were washed in ice-cold PBS and pelleted by centrifugation at 400 × g for 5 minutes. The pellet was resuspended in 100 *μ*l of lysis buffer (250 mM sucrose, 70 mM KCl, 0.5 mM DTT, 100 *μ*M PMSF, 2 *μ*g/ml pepstatin, 25 *μ*M ALLN, 2.5 *μ*g/ml aprotinin, 10 *μ*M leupeptin, and 2 mg/ml digitonin). After 5 minutes incubation on ice, the samples were centrifuged for 5 minutes at 20,000 × g. The supernatant (cytosolic fraction) was carefully removed, and the pellet (mitochondrial fraction) was resuspended in lysis buffer. 

### 2.9. Measurement of Mitochondrial Transmembrane Potential (*∆*Ψm)

Changes in *∆*Ψm were detected using tetramthylrhodamine ethyl ester perchlorate (TMRE) (Molecular Probes). Trypsinized cells were combined with supernatant medium and incubated with TMRE (100 nM) for 30 minutes at room temperature in the dark. TMRE fluorescence was measured using the FL2 channel (582 nm) of FacsCalibur flow cytometer (Becton Dickinson). A 45-minute CCCP (10 *μ*M) treatment was used to uncouple mitochondria, as a positive control. 

## 3. Results and Discussion

### 3.1. Prolonged ER Stress Induces Apoptosis and Mitochondrial Membrane Depolarization

**Figure 1:**
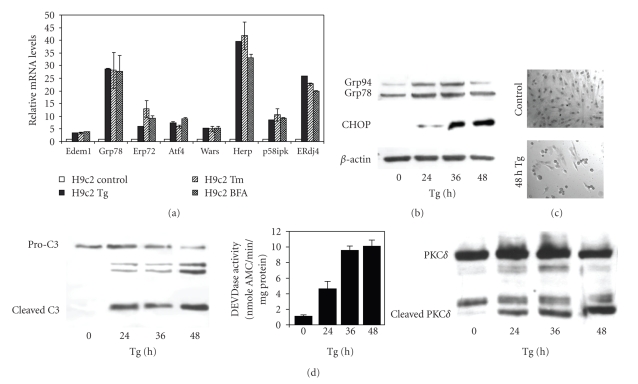
*ER stress-induced apoptosis in H9c2 cells.* (a) H9c2 cells were left untreated or treated with (2 *μ*M) Tg, (2 *μ*g/ml) Tm or (2 *μ*g/ml) BFA for 24 hours. The change in expression levels of ER stress markers was measured by real-time RT-PCR normalizing against *GAPDH* expression and plotting expression levels relative to the control. Error bars represent mean ± SD from an experiment performed in duplicate and reproduced twice. (b) H9c2 cells were left untreated or treated with (2 *μ*M) Tg for the indicated times. The induction of ER stress markers, Grp78, Grp94 and CHOP was determined by Western blot analysis. *β*-actin was used to determine equal loading of samples. (c) H9c2 cells were left untreated or treated with (2 *μ*M) Tg for the indicated times. After 48 hours of Tg-treatment, cells were stained with haematoxylin-eosin-stain and photographed at 200 × magnification. (d) H9c2 cells were left untreated or treated with (2 *μ*M) Tg for the indicated times. The processing of procaspase-3 and cleavage of PKC*δ* were determined by Western blot analysis. The caspase activity was determined using DEVD-AMC. The figure is a representative of three independent experiments.

To induce ER stress, H9c2 cells, a neonatal rat cardiomyocyte-derived cell line, were treated with three different ER stress-inducing agents: thapsigargin (Tg) an inhibitor of the Sacroplasmic/endoplasmic reticulum Ca^2+^-ATPase (SERCA) pump, tunicamycin (Tm) an inhibitor of N-linked glycosylation, and brefeldin A (BFA) an inhibitor of protein transfer from the ER to the Golgi. Treatment of H9c2 cells with any of these ER stress inducing agents caused an increase in the mRNA levels of many genes associated with the ER stress response (Figure 1(a)). We also examined protein levels by Western blot analysis for a subset of these genes and found them to reflect the changes observed in mRNA expression with Grp78, Grp90, and the proapoptotic transcription factor CHOP/GADD153 being significantly upregulated after treating cells with Tg (Figure 1(b)). Conditions of prolonged (48 hours) ER stress induced morphological changes associated with apoptosis, including cellular shrinkage, nuclear condensation, and membrane blebbing (Figure 1(c)). ER stress-induced apoptosis was associated with activation of caspases as detected by the processing of caspase-3, an increase in DEVDase activity, and the cleavage of protein kinase C delta (PKC*δ*)*,* a cellular substrate of caspases, which was detectable as early as 24 hours post Tg treatment (Figure 1(d)). Collectively, these data demonstrate that ER stress induces apoptotic cell death in H9c2 cardiomyocytes. 

**Figure 2:**
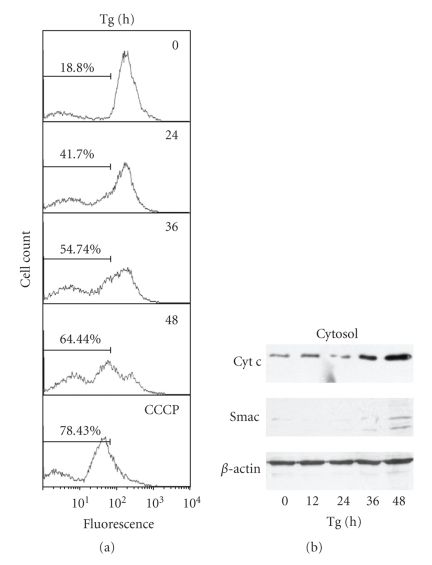
*Loss of mitochondrial membrane potential and release of cytochrome c during ER stress-induced apoptosis.* (a) H9c2 cells were treated with (2 *μ*M) Tg for the indicated times. Following treatment, cells were incubated with (100 nM) TMRE. Mitochondrial membrane potential was monitored by measuring the fluorescence intensity at 582 nm (FL2). As a positive control for depletion of membrane potential, cells were treated with (10 *μ*M) CCCP for 45 minutes. The data is a representative of at least three independent experiments. (b) Western blot analysis of cytochrome *c* and Smac in cytosolic fractions. The data is a representative of at least three independent experiments.

Several reports have indicated a possible role for mitochondria in ER stress-induced apoptosis [10, 27]. To determine the exact contribution of the mitochondria to ER stress-induced apoptosis, alterations of ΔΨm and release of mitochondrial intermembrane space proteins into the cytosol were analyzed. Alterations in ΔΨm were studied using tetramethyl rhodamine ethyl ester (TMRE). The loss of ΔΨm was detectable by flow cytometry starting after 24 hours of induction of ER stress and increasing over time (Figure 2(a)). Consistent with a drop in ΔΨm, Western blot analysis demonstrated increased cytosolic levels of cytochrome *c* and Smac, when compared to untreated controls, at 36 and 48 hours post Tg treatment (Figure 2(b)). The slight differences observed in the kinetics of release from mitochondria between cytochrome *c* and SMAC may be due the differences in the affinity of cytochrome *c* and SMAC antibodies used for western blotting. These results show that ER stress induces MOMP and the release of proapoptotic proteins from the intermembrane space into the cytosol. These observations suggest that the loss of ΔΨm and release of mitochondrial intermembrane space proteins into the cytosol are coupled and a component of ER stress-induced apoptosis.

### 3.2. Effect of Caspases on ER Stress-Induced Drop in ΔΨm

**Figure 3:**
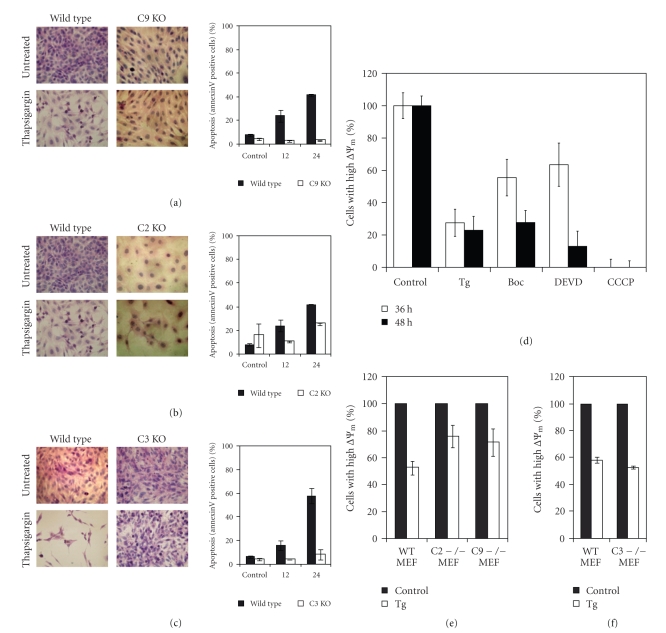
*Resistance to ER stress-induced death and loss of mitochondrial membrane potential in absence of caspases.* (a)–(c) *Left panel*, Indicated MEFs were treated with (2 *μ*M) Tg for 24 hours. Cells were stained with haematoxylin-eosin-stain and visualised using an Olympus IX71 microscope at 40×. Images are representative of 2 independent experiments. *Right panel*, indicated MEFs were treated with (2 *μ*M) Tg for the indicated times. Increase in cell death was measured by annexin V staining. The data is representative of at least 2 independent experiments. (d) H9c2 cells were treated with (2 *μ*M) Tg alone or pretreated for 30 minutes with Boc-D.fmk (20 *μ*M) and DEVD.fmk (20 *μ*M) prior to treatment with Tg, for the indicated time periods. Following treatment cells were incubated with (100 nM) TMRE. Mitochondrial membrane potential was monitored by measuring the fluorescence intensity at 582 nm (FL2). As a positive control for depletion of membrane potential, cells were treated with (10 *μ*M) CCCP for 45 minutes. The data is a representative of at least three independent experiments. (e)–(f) Indicated MEFs were treated with (2 *μ*M) Tg for 24 hours. Following treatment cells were incubated with (100 nM) TMRE. Mitochondrial membrane potential was monitored by measuring the fluorescence intensity at 582 nm (FL2). The data is a representative of at least three independent experiments.

In Tg-treated H9c2 cells, we detected caspase-3 activation starting as early as 12–18 hours, which preceded detectable changes in the mitochondria. This suggested a possible involvement of caspases in inducing ΔΨm depletion. Caspase-3 and -9 are important in both the intrinsic and extrinsic pathways of apoptosis. To determine the role of these caspases in ER stress-induced cell death, we have used mouse embryonic fibroblasts (MEFs) deficient in caspase-3, -2, and -9. Fibroblasts from wild-type and homozygous knock-out embryos were treated with 2 *μ*M Tg for 24 hours, and MEFs were assayed for viability using annexin V staining. As shown in Figures 3(a)–3(c), caspase-9, -2, and -3 knock-out MEFs were protected against apoptosis induced by Tg. Next we tested the effect of broad range caspase inhibitors on the ER stress-mediated drop in *∆*Ψm. H9c2 cells were treated with Tg in the presence or absence of the caspase inhibitors Boc-D.fmk and DEVD.fmk (Figure 3(d)). Inhibition of caspases reduced Tg-induced loss of ΔΨm up to 36 hours, but loses effectiveness at 48 hours. To further confirm the role of caspases in ER stress-mediated ΔΨm loss, we determined mitochondrial membrane potential in caspase-9, -2, and -3 deficient and wild-type MEFs upon exposure to ER stress. We observed that caspase-9 and caspase-2 deficient MEFs showed resistance to ER stress-mediated loss of ΔΨm as compared to wild-type MEFs (Figure 3(e)). In contrast, caspase-3 deficient MEFs showed loss in ΔΨm comparable to wild-type MEFs (Figure 3(f)). These results suggest that caspase-2 and -9 but not caspase-3 play a role in the ΔΨm loss during ER stress-induced apoptosis. The apparent differences in ER stress-mediated loss of ΔΨm upon DEVD.fmk pretreatment (Figure 3(d)) and in caspase-3 deficient MEFs (Figure 3(f)) may be due to inhibition of both caspase-3 and -7 by DEVD.fmk. This is in agreement with a previous study that showed that early apoptoptic events (e.g., Bax translocation and cytochrome *c* release) following mitochondria-mediated apoptosis triggered by UV irradiation were compromised by a double knock-out of caspase-3 and -7 in MEFs, but not in caspase-3 knock-out cells [30]. 

**Figure 4:**
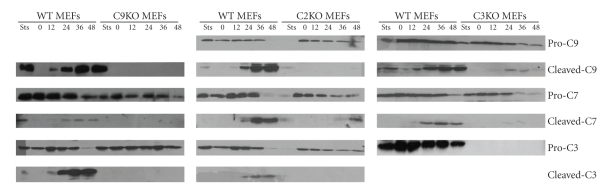
*Altered caspase processing in caspase-9, -2, or -3 deficient MEFs upon exposure to ER stress.* Indicated wild-type and caspase deficient MEFs were treated with thapsigargin (2 *μ*M) for 12, 24, 36, and 48 hours. Protein extracts were isolated and subjected to western blot analysis using the indicated antibodies. Staurosporine (Sts) 100 nM for 12 hours was used as a positive control for caspase processing. Images are representative of a least 2 independent experiments.

To determine the functional consequences of loss of caspase-9, -2, and -3 in ER stress-induced apoptosis, we characterized the processing of pro-caspase-3, -7, and -9 in wild-type and corresponding knock-out MEFs by Western blotting of whole cell lysates after treatment with Tg (Figure 4). In wild-type cells, processing of pro-caspase-3, -7, and -9 was observed upon Tg treatment as compared with untreated controls (Figure 4). Processing of pro-caspase-3 and -7 was, however, completely inhibited in caspase-9 knock-out MEFs. This indicates that proteolytic activation of pro-caspase-3 and -7 during ER stress-induced apoptosis is dependent on caspase-9. Further, pro-caspase-9 processing was strongly reduced in the caspase-3 knock out MEFs (Figure 4). The activated effector caspase-3 acts on caspase-9 processing in a feedback amplification loop that results in complete activation of caspase-9, and consequently loss of caspase-3 may prevent complete activation of pro-caspase-9 [31]. Recently, it has been shown that caspase-2 can serve as a proximal caspase that functions upstream of mitochondria during ER stress-induced apoptosis, cleaving the BH3-only protein Bid which then functions as a critical apoptotic switch [9]. In this study the cleavage of caspase-3 and -9 was inhibited in caspase-2 knock-out MEFs, which corroborates the importance of this caspase in ER stress-induced apoptosis. However, we observed that there was some processing of caspase-7 in caspase-2 deficient MEFs. In agreement with these results, we observed that resistance to ER stress-induced cell death in caspase-2 deficient MEFs was not as pronounced as in caspase-9 deficient MEFs (Figures 3(a) and 3(b)). However, at present the mechanism for processing of caspase-7 in caspase-2 deficient MEFs is not clear. Taken together, the data from whole cell lysates indicates that caspase-2 and -9 play an important role in Tg-induced apoptosis. 

### 3.3. The Role of Bcl-2 Family Proteins in ER Stress-Induced Drop in ΔΨm

**Figure 5:**
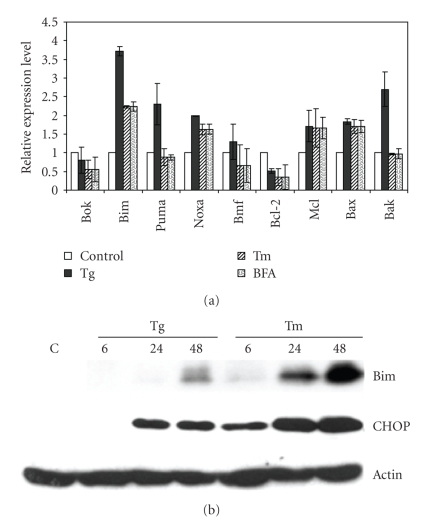
*ER stress-mediated induction of Bcl-2 family members in H9c2 cells.* (a) H9c2 cells were left untreated or treated with (2 *μ*M) Tg, (2 *μ*g/ml) Tm, or (2 *μ*g/ml) BFA for 24 hours. The change in expression levels of ER stress markers was measured by real-time RT-PCR normalized against *GAPDH* expression and plotting expression levels relative to the control. Error bars represent mean ± SD from an experiment performed in duplicate and reproduced twice. (b) H9c2 cells were left untreated or treated with (2 *μ*M) Tg, (2 *μ*g/ml) Tm for indicated time points, and induction of Bim, and CHOP was determined by western blot analysis. *β*-actin was used to determine equal loading of samples.

**Figure 6:**
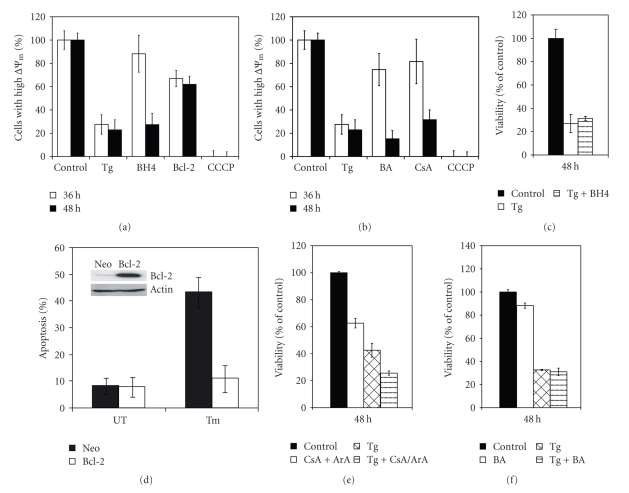
*Regulation of ER stress-induced mitochondrial changes by Bcl-2 family proteins.* (a) H9c2 cells were treated with (2 *μ*M) Tg alone or pretreated for 30 minutes with BH4-Tat peptide (200 nM) prior to treatment with Tg, for the indicated time periods. Bcl-2 overexpressing H9c2 cells were treated with Tg (2 *μ*M) for the indicated time periods. (b**)** H9c2 cells were treated with Tg (2 *μ*M) alone or pretreated for 30 minutes with bongkrekic acid (BA) (10 *μ*M); cyclosporine A (CsA) (2 *μ*M) and aristolochic acid (ArA, Calbiochem) at 25 *μ*M prior to treatment with Tg, for the indicated time periods. (a)-(b) Following treatment, cells were incubated with TMRE (100 nM). Mitochondrial membrane potential was monitored by measuring the fluorescence intensity at 582 nm (FL2). As a positive control for depletion of membrane potential, cells were treated with 10 *μ*M CCCP for 45 minutes. The data is a representative of at least three independent experiments. (c) H9c2 cells were treated with (2 *μ*M) Tg alone or pretreated for 30 minutes with BH4-Tat peptide (200 nM) prior to treatment with Tg, for 48 hours and reduction in cell viability was determined by MTT assay. Error bars represent mean ± SD from three independent experiments performed in triplicates. (d) Bcl-2 overexpressing H9c2 cells (Bcl-2) and control cells (neo) were treated with Tm (2 *μ*g/ml) for 48 hours. Increase in cell death was measured by annexin V staining. The data is representative of at least 3 independent experiments. Protein extracts from Bcl-2 overexpressing H9c2 cells (Bcl-2) and control cells (neo) were subjected to western blot analysis using the indicated antibodies. (e)-(f) H9c2 cells were treated with Tg (2 *μ*M) alone or pretreated for 30 minutes with (e) cyclosporine A (CsA) (2 *μ*M) and aristolochic acid (ArA) at 25 *μ*M; (f) bongkrekic acid (BA) (10 *μ*M); prior to treatment with Tg, for 48 hours and reduction in cell viability was determined by MTT assay. Error bars represent mean±SD from three independent experiments performed in triplicates.

The mitochondrial apoptotic signalling pathway involves activation of the proapoptotic Bcl-2 family members Bax and Bak, that induce permeabilization of the mitochondrial outer membrane and release of cytochrome *c* [18]. To investigate the involvement of Bcl-2 family proteins in ER stress-induced cell death, we determined the effect of ER stress on the expression levels of Bcl-2 family members in H9c2 cells (Figure 5(a)). Our studies demonstrated that while Noxa, Mcl1, and Bax were upregulated by all three ER stress-inducing agents (Tg, Tm and BFA), Bim showed the greatest fold changes in response to any type of ER stress. The upregulation of Bim protein upon ER stress was confirmed by Western blotting (Figure 5(b)). We observed that Tg was most effective in inducing Bim mRNA levels; however, Tm was more potent in inducing Bim protein levels. This could be due to the posttranslational modifications regulating BIM protein stability upon exposure to ER stress [22]. In order to test the function of Bcl-2 on the mitochondria, we used the cell permeable BH4-Tat peptide. The BH4 domain of antiapoptotic Bcl-2 family members accumulates on the mitochondria and inhibits cell death [32]. Pretreatment of cells with BH4-Tat protected the mitochondria against the effect of Tg, delaying membrane depolarisation by at least 12 hours (Figure 6(a)). Next we generated a Bcl-2 overexpressing H9c2 clone and examined the protective potential of Bcl-2 in these cells. Bcl-2 overexpression was able to prevent loss of ΔΨm upon Tg treatment, for at least 48 hours (Figure 6(a)). Furthermore, Bcl-2 overexpression efficiently protected cells against Tm induced cell death (Figure 6(d)), whereas pretreatment of cells with BH4-Tat was not able to inhibit ER stress-induced apoptosis (Figure 6(c)). 

### 3.4. The Role for Mitochondrial Permeability Transition Pore in the ER Stress-Induced Drop in ΔΨm

Next we evaluated whether MPT was involved in ER stress-induced loss of mitochondrial membrane potential. For this purpose, we used two PTP inhibitors, bongkrekic acid and a combination of cyclosporine A and aristolochic acid. A 30-minute pretreatment with either the combination of 2 *μ*M cyclosporine A (inhibitor of cyclophylin D) and aristolochic acid (25 *μ*M) or 10 *μ*M bongkrekic acid (an inhibitor of the adenine nucleotide transporter (ANT)), prior to Tg treatment prevented loss of ΔΨm at 36 hours (Figure 6(b)). This protective effect was lost by 48 hours, suggesting limited efficacy of the drugs or a contribution of PTP independent processes. In line with the transient effect on ΔΨm, pretreatment of the cells with either the combination of 2 *μ*M cyclosporine A and aristolochic acid (25 *μ*M) or 10 *μ*M bongkrekic acid was not able to inhibit ER stress-induced apoptosis (Figures 6(e) and 6(f)). 

In this study, we have investigated the factors regulating the loss of mitochondrial membrane potential during ER stress-induced apoptosis. Involvement of the mitochondria during ER stress-induced apoptosis seems to be a central amplification step and probably a point of no return [18]. The majority of cells are committed to die following MOMP because it leads not only to the activation of the well-established caspase-mediated apoptotic pathway, but, should there be a failure of its execution through insufficient caspase activation, a parallel, caspase-independent cell death pathway is set in motion that is controlled by HtrA2/Omi, AIF and Endo G [33, 34]. Although a number of mechanisms may be responsible for ER stress-induced mitochondrial changes, caspase activation upstream of the mitochondria has been linked to ΔΨm depolarization [7, 30]. We used MEFs deficient in caspase-3, -2, or -9 in order to determine the roles of these proteases in the ER stress apoptotic program, and subsequently established a role for these proteases in ER stress-induced apoptosis. Our results show that caspases are activated during ER stress, with caspase-2 and -9 acting upstream of caspase-3 and -7. 

The Bcl-2 protein family governs mitochondrial homeostasis. Besides the mitochondria, Bcl-2 proteins are also localised at the ER [6]. However, the primary site of their action and the exact mechanism by which they control cell fate during ER stress was not fully understood. Here we show that overexpression of wild type Bcl-2 is able to protect mitochondria from the effect of Tg. A restricted, but similar protective effect was seen by transducing the BH4 domain of Bcl-2 into H9c2 cells. The BH4 peptide has been shown to localise at the mitochondria, suggesting that mitochondrial localised antiapoptotic Bcl-2 proteins are able to prevent ER stress-induced mitochondrial damage [32]. Besides their effect on the PTP, multidomain proapoptotic Bcl-2 proteins in the outer mitochondrial membrane can oligomerize to form nonspecific conducting channels through which cytochrome *c* can be released [6]. During conditions of ER stress, BH3-only proteins are activated either by transcriptional upregulation or through posttranslational modifications. Once activated, the BH3-only proteins converge on the activation of multidomain proapoptotic proteins Bax or Bak, which act as a gateway to the intrinsic apoptotic pathways operating at the mitochondria [18]. Recently, ER stress was shown to upregulate Bim through CHOP-C/EBP*α*-mediated direct transcriptional induction [22]. Therefore, upregulation of BH3-only proteins, such as Bim, at the transcriptional level may result in activation of Bax/Bak at the mitochondria, triggering cell death. The protective effect of Bcl-2 supports a role for proapoptotic Bcl-2 family members in targeting the mitochondria upon ER stress. In conclusion, our results show that multiple signals such as caspase activation and induction of BH3-only proteins converge on the mitochondria upon induction of ER stress and these signals trigger MOMP and loss of mitochondrial membrane potential.
